# Parent-teacher discrepancies in externalizing behavior predicting social skills development in middle childhood

**DOI:** 10.1016/j.appdev.2025.101856

**Published:** 2025-08-27

**Authors:** Taehee Kim, Kristen L. Bub, Sadona K.R. Thompson

**Affiliations:** Department of Educational Psychology, University of Georgia, USA

**Keywords:** Social skills, Externalizing behavior, Multi-group latent growth model, Informant discrepancy, Middle childhood

## Abstract

This longitudinal study examined parent-teacher agreement and discrepancies in externalizing behavior assessments and their association with children’s social skill development. Using third-grade externalizing behavior reports, 927 students were categorized into four groups: both informants reporting clinical concerns, only teacher reporting clinical concerns, only parent reporting clinical concerns, and both reporting normative levels. A multi-group latent growth model analyzed social skill trajectories from third through sixth grade. Results indicated that 21% of students fell within discrepancy groups, where parents and teachers reported different levels of behavior severity. These groups included a higher proportion of students from non-White and low socioeconomic status backgrounds. Students in discrepancy groups showed more adaptive social skill trajectories than peers with clinical concerns reported by both informants but exhibited less favorable trajectories compared to those with normative behavior reports across contexts. Notably, students identified by teachers as demonstrating elevated externalizing behaviors showed significant linear growth in school-based social skills over time, suggesting a potential impact of teacher-initiated interventions. Findings highlight the importance of a multi-informant approach in accurately understanding and supporting students’ social development, as parent and teacher perspectives provide complementary information about children’s behavioral functioning across contexts.

Social skills in middle childhood are fundamental for building competencies that affect children’s social functioning, academic performance, and overall well-being (Blandon, Calkins, Grimm, Keane, & O’Brien, 2010; Caemmerer & Keith, 2015; Ziv, Goldin, Jazaieri, Hahn, & Gross, 2013). Supporting children in developing these skills and providing ample opportunities for positive social interactions is therefore essential. However, heightened levels of externalizing behaviors, such as aggression and deviant behaviors, have been associated with difficulties in social development (Hukkelberg, Keles, Ogden, & Hammerstrom, 2019). These behaviors, characterized by outward expressions of defiance and hostility, place children at risk of social rejection, limiting their opportunities for peer interaction and further compromising their social development (Godleski, Kamper, Ostrov, Hart, & Blakely-McClure, 2015). Given the potential for externalizing behavior to act as a risk factor in later social skills development, early identification and intervention in externalizing behaviors has been highlighted (Hart, Maharaj, & Graziano, 2019; Schindler et al., 2015). To accurately identify children in need, a multi-informant approach—incorporating perspectives from various sources, such as parents and teachers—is often used (Hunsley & Mash, 2018). While this approach enhances the comprehensiveness and accuracy of behavioral assessments, informants frequently provide differing evaluations of the assessed behaviors in children (i.e., informant discrepancy; Achenbach, McConaughy, & Howell, 1987; De Los Reyes et al., 2015). In the past, these discordant ratings have been regarded as measurement error or, in some cases, have been overlooked when multi-informant approaches are not implemented across settings (De Los Reyes et al., 2022).

However, a growing body of literature has demonstrated that discrepancies in ratings between informants often serve as crucial indicators of context-specific risks, providing valuable information about children’s functioning across different settings (Achenbach, 2016; De Los Reyes et al., 2015; Martel, Markon, & Smith, 2017). Recent clinical evaluation practices also emphasize the importance of integrating informant discrepancies into assessment processes for a more comprehensive understanding of children’s behavior and individualized treatment (De Los Reyes & Epkins, 2023). Despite the value and the frequent occurrence of informant discrepancies, developmental research incorporating these discrepancies in ratings remains limited. Drawing on prior studies demonstrating the influence of early behavioral problems on social skill development, our study seeks to expand this understanding by examining how discrepancies in early behavioral assessments can affect later developmental trajectories. Specifically, we aimed to investigate how parent-teacher agreement and disagreement regarding the level of externalizing behaviors predict social skill trajectories in middle childhood.

## Social skills development in middle childhood

Social skills encompass learned behaviors that individuals exhibit to successfully complete a social task (Gresham, 2016). The development of social skills in youth is pivotal as it lays the foundation for successful relationships with others, academic achievements, and mental well-being (Blandon et al., 2010; Caemmerer & Keith, 2015; Ziv et al., 2013). In particular, social skills acquired in middle childhood can serve as foundational assets for later social adjustment in adolescence, promoting smoother behavioral and social adjustment within school contexts (Domitrovich, Durlak, Staley, & Weissberg, 2017) and bolstering social support systems that help alleviate psychological distress (Segrin, McNelis, & Swiatkowski, 2016). Despite the recognized importance of social skills in child development, studies examining their developmental trajectories in middle childhood remain limited and yield inconsistent results (Sørlie, Hagen, & Nordahl, 2021). For example, DiDonato (2014) observed mean-level growth in interpersonal skills from kindergarten through fifth grade, identifying two groups: one with a higher, primarily stable level and another with a moderate level that exhibited a slight increase over time. Côté, Tremblay, Nagin, Zoccolillo, and Vitaro (2002) identified three distinct groups—high, medium, and low—which all displayed stability in social skill growth from kindergarten through sixth grade. In contrast, Sørlie et al. (2021) found that while the majority of students from fourth to seventh grade maintained stable average scores over time (72%), two smaller groups (14% each) showed diverging patterns, with one group’s scores declining from a high baseline and the other’s increasing from a lower baseline. Sallquist et al. (2009), however, reported a mean-level decrease in perceived social competence from kindergarten to early adolescence. Hajovsky, Caemmerer, and Mason (2022) further noted gender differences, with boys showing a slight decline in social skills from kindergarten through sixth grade, while girls consistently demonstrated significantly higher and stable social skills at each assessment point. Despite variations in analytic methods and samples across studies, these mixed findings highlight the need for continued research into social skills trajectories during this formative period. Given these inconsistent findings, the present study aims to enhance our understanding of social skills development during middle childhood by examining how distinct parent-teacher perspectives on externalizing behaviors are associated with subsequent social skills trajectories.

## Early externalizing behavior and social skills development

Early behavioral problems can significantly heighten the risk of children encountering social challenges and developing maladaptive social trajectories. Extensive research demonstrates that externalizing issues, such as aggression and disruptive behaviors, are closely tied to difficulties in peer relationships and hinder the development of social skills (Chen, Huang, Chang, Wang, & Li, 2010; Hukkelberg et al., 2019; Racz, Putnick, Suwalsky, Hendricks, & Bornstein, 2017). For instance, aggressive children often experience peer rejection or victimization (Godleski et al., 2015), limiting their opportunities to engage in positive social interactions crucial for acquiring essential social skills. These social consequences may be particularly pronounced during middle childhood, when externalizing behaviors that were considered developmentally normative in early childhood become less socially acceptable (Roskam, 2019). Longitudinal evidence further supports the link between behavioral problems and social skills development (Racz et al., 2017), due to the cyclical nature of early behavioral risks and the maladaptive social interactions they experience (Blandon et al., 2010). This recursive relationship is often conceptualized as a *developmental cascade* wherein issues in one domain affect adaptive functioning in another over time (Masten & Cicchetti, 2010). Chen et al. (2010) tracked students from third to sixth grade and found that aggression at each assessment influenced subsequent peer relationships. The authors suggested that aggressive behavior fosters negative attitudes toward peers and school, undermining children’s ability to engage in positive social interactions.

Two studies have specifically examined the effects of early externalizing behavior on subsequent social skill development in middle childhood. Berry and O’Connor (2010) found that children with high levels of internalizing behaviors (e.g., anxiety, depression) in preschool demonstrated less optimal social skills from kindergarten through sixth grade compared to those with lower levels of internalizing behavior, but externalizing behaviors were not significantly associated with later social skill trajectories. In contrast, Sorlie et al. (2021) found that classroom externalizing behaviors in third grade, such as physical aggression and rule-breaking, had a negative impact on social skills development from fourth to seventh grade. This was especially true for children who began with average social skills that remained stable over time, and those who started with lower social skills but had been gradually improving. While the theoretical framework of developmental cascades and substantial empirical evidence suggest a strong link between externalizing behaviors and social skills, these conflicting findings indicate that additional longitudinal research can further illuminate the effects of early externalizing behaviors on subsequent social skills development.

## Informant discrepancies in externalizing behaviors between parents and teachers

Identifying externalizing behaviors at an early stage allows for timely intervention, which can disrupt maladaptive developmental trajectories and foster healthier pathways (Hart et al., 2019; Schindler et al., 2015). Efforts to detect externalizing behavior problems include the use of a multi-informant, multi-method, multi-setting assessment approach, which provides a more comprehensive understanding of children’s behavioral problems across different contexts (e.g., home, school) from the perspective of different individuals (e.g., parent, teacher) through the use of different methods (e.g., rating scales, interviewing; Hunsley & Mash, 2018). For school-aged children, parents and teachers serve as primary informants to capture behaviors in home and school settings, respectively. However, one challenge is the low agreement between parents’ and teachers’ reports of the child’s behavior. This phenomenon, known as *informant discrepancies*, consistently arises when multiple informants are involved in psychological and behavioral assessments. Several meta-analyses have consistently shown low levels of agreement between informants on externalizing behavior problems (Achenbach et al., 1987; De Los Reyes et al., 2015; Huang, 2017).

Over the past two decades, informant discrepancies in child behaviors have gained increasing theoretical and empirical support as valuable indicators of contextual variations in behavior. Parents and teachers, as representative observers in different contexts, provide unique perspectives that can enhance our understanding of children’s behaviors and the interactive environments in which they occur (Achenbach, 2016; Martel et al., 2017). Within the Operations Triad Model, De Los Reyes, Thomas, Goodman, and Kundey (2013) conceptualized informant discrepancies as encompassing both measurement errors (i.e., Compensating Operations) and meaningful differences that provide context-specific information about child behaviors and environments (i.e., Diverging Operations). For example, elevated aggression levels observed at school may be attributed to measurement errors; however, they might also indicate genuine variations in child behaviors and contextual factors that initiate or sustain these behaviors in that specific setting. Middle childhood is a stage in which both parents and teachers remain key informants of children’s behavior. Yet, as social demands become more complex (e.g., increased expectations for collaboration, adherence to group norms), behavior may manifest differently across home and school settings. These contextual shifts make middle childhood a particularly important period for examining informant discrepancies in externalizing behavior and how such discrepancies relate to later social skill development.

## Informant discrepancies and subsequent development

Building on the understanding that informant discrepancies in behavioral problems can be used as tools for understanding the current behaviors and contexts of children, researchers also posit that these discrepancies can help to predict future developmental and clinical outcomes (De Los Reyes & Epkins, 2023). Despite limited research specifically focusing on the predictive value of parent-teacher discrepancies, empirical evidence from studies on parent-youth discrepancies indicates that informant discrepancies may have predictive utility (e.g., Augenstein et al., 2022; Goolsby et al., 2018). Laird and De Los Reyes (2013) found that agreement and disagreement between informants uniquely contributed to explaining aspects of child psychopathology. For example, antisocial behaviors were most pronounced when both adolescents and parents agreed that the adolescent frequently engaged in rule-breaking behaviors (e.g., break a rule, get into trouble). In contrast, the highest levels of depression were observed when adolescents reported high levels of rule-breaking while parents reported low levels. Additionally, several studies suggest that informant discrepancies can predict treatment outcomes, with greater parent-child disagreement often associated with reduced intervention effectiveness (Becker-Haimes, Jensen-Doss, Birmaher, Kendall, & Ginsburg, 2018; Hennig, Schramm, & Linderkamp, 2018). For example, Becker-Haimes et al. (2018) found that when parents reported more anxiety symptoms than their children at the beginning of treatment, youth were less likely to achieve diagnostic remission following cognitive behavioral therapy. Goolsby et al. (2018) emphasized that addressing pre-treatment discrepancies can be a critical component of the therapeutic process to improve outcomes.

Although limited research has directly examined the predictive value of parent-teacher discrepancies in child behavior for developmental outcomes such as social skills and emotional wellbeing, there is evidence to suggest that such discrepancies may shape children’s environments in developmentally important ways. When teachers and parents hold differing views about a child’s abilities or needs, it can escalate conflicts between them (Lake & Billingsley, 2000). Lasater (2016) demonstrated that disagreements concerning children’s academic and social competencies sometimes led teachers to avoid conversations with parents, engage in negative interactions, or even develop fear of the parents themselves. As a result, such conflicts often remain unresolved, potentially undermining the development of supportive partnerships for positive child development. Furthermore, teachers and parents are not merely detached observers; they actively shape children’s social behaviors through the expectations and norms in each context (Farmer, Lines, & Hamm, 2011). Differences in reporting may reflect different behavioral expectations between home and school settings (De Los Reyes & Epkins, 2023), which may further complicate a child’s developmental progress by creating confusion about behavioral norms. Carter and Pool (2012) noted that inconsistent behavioral expectations across contexts can contribute to challenging behaviors in young children. Moreover, as parents and teachers are the primary informants in the referral processes for interventions and services, their discrepancies in reporting could significantly influence the type of care and developmental support children receive.

Despite growing evidence supporting the interpretative and predictive utility of informant discrepancies, developmental research has insufficiently leveraged these discrepancies to understand a child’s social-behavioral development. Our study addresses this research gap by examining how discrepancies between parent and teacher reports of externalizing behaviors predict social skill trajectories during middle childhood. Considering that parent-teacher discrepancies are also prevalent in social skills (Kuhfeld, Soland, & Lewis, 2023) and that teacher- and parent-reported social skills reflect unique perspectives shaped by their respective contexts (school vs. home), we analyzed both teacher and parent reports of social skills separately. By examining how discrepancies in externalizing behaviors between teachers and parents predict social skill development within each context, this approach contributes to a more comprehensive understanding of how informant discrepancies can be utilized to improve developmental outcomes across settings.

## The current study

Our study examines how parent-teacher agreement and discrepancy in evaluating children’s externalizing behaviors in third grade predict social skill development trajectories through sixth grade. Third grade represents a critical period, as children are expected to navigate increasingly complex academic and social demands in school, and peer relationships become more central to their social development. Externalizing behaviors during this stage can act as barriers to forming and maintaining social relationships, highlighting the importance of evaluations from both parents and teachers to identify children exhibiting heightened behavioral issues that may require clinical consideration. However, parents and teachers often provide differing evaluations across home and school contexts, which can affect the identification and support of students in need of intervention. To better understand the implications of these parent-teacher discrepancies in behavior ratings on social development, we address two primary objectives in this study. First, this study explores social skills trajectory from third through sixth grade to establish general developmental patterns during this period before introducing informant discrepancies as a new layer of analysis. Second, the study investigates how parent-teacher agreement and discrepancies regarding externalizing behaviors predict variations in social skill trajectories. This analysis employs a multi-informant approach, examining two separate sets of models: one utilizing teacher-reported social skills and another utilizing parent-reported social skills. By focusing on the unique variance explained by informant discrepancies and agreements, we aim to deepen our understanding of how early differences in behavioral evaluations may signal distinct developmental pathways in social skills.

## Method

### Participants

This study utilized data from Phases I and III of the NICHD Study of Early Child Care and Youth Development (SECCYD), which is one of the most comprehensive longitudinal studies of children and their developmental environments (for more information visit https://www.icpsr.umich.edu/web/ICPSR/series/00233). Phase I initially enrolled 1364 children and their families across 10 geographical locations, with recruitment occurring shortly after birth in hospitals and enrollment contingent upon completion of the one-month visit. Phase III began when participating children entered second grade, with 1061 children and their families remaining in the study. In Phase III, data were collected using social age (i.e., years in school) rather than chronological age (i.e., age in months or years) to better reflect social expectations around behavior (e.g., aggression or cooperation) and relationships (e. g., peer dynamics). These participants were then tracked continuously through sixth grade. The current analytic sample comprised 927 students (49% boys, 51% girls) of the original 1061 children and families in Phase III. For this study, participants were tracked from third to sixth grade (typically around ages 8–12 years). All participants included in the analysis had both parent and teacher reports of externalizing behaviors at third grade, as well as data for at least one time point of social skills assessment (from either parent or teacher) across the four measurement periods (Grades 3, 4, 5, and 6). This ensured that all participants contributed meaningful data to the analysis without having missing values for all variables in our model. Of the respondents, approximately 82.4% were White, and the remaining 17.6% of respondents were ethnic minority children (11% Black, 1.3% Asian or Pacific Islander, 0.3% American Indian or Alaska Natives, 5% Other Ethnicity). The average income-to-needs was 4.4 times the poverty line and 76.5% of families were living-above-poverty; 16.5% of families were living-near-poverty; 7% were living-in-poverty.

### Procedures

The data for the current study were collected through reports provided by both teachers and parents. Parental reports were used to determine the child’s sex and race/ethnicity when the child was one month old. Family income information was collected when the children were in third grade, and an income-to-needs ratio was calculated. Parent and teacher reports of externalizing behaviors were obtained through questionnaires (i.e., *Child Behavior Checklist*) when children were in third grade. Assessments of social skills were conducted annually over four years, from third (Time 1) to sixth grade (Time 4), with both parents and teachers using instruments that featured parallel forms for parent and teacher (i.e., *Social Skills Rating System)*. For more information about the data collection procedures, please refer to the SECCYD data archive (https://www.icpsr.umich.edu/web/ICPSR/series/00233). This study involved secondary analysis of de-identified data and was reviewed and determined to be exempt by Institutional Review Board of University of Georgia (PROJECT00007243).

### Measures

#### Externalizing Behavior

Externalizing Behavior was assessed by the *Child Behavior Checklist* (CBCL; Achenbach, 1991a) for parent reports and *Teacher Report Form of the Child Behavior Checklist* (TRF; Achenbach, 1991b) for teacher reports. The CBCL was designed to obtain parents’ reports on their child’s behavior problems and functioning. The TRF, derived from the CBCL, captures teacher observations of behavioral problems. Teachers and parents were asked to rate how well item statements describe the behavior of the child now or within the past six months using 3-point scales from 0 = “*not true of the child*” to 2 = “*very true of the child*”. Externalizing Problems are made up of two syndrome scales, which are Aggressive (e.g., Argues a lot) and Delinquent Behaviors (e.g., Lying or Cheating), and they identify disruptive, antisocial, and impulsive behavior. The CBCL includes 33 items and the TRF includes 34 items for assessing Externalizing Problems. Although many items are identical across the two forms (e.g., Gets in many fights), each also includes items tailored to capture context-specific behaviors (e.g., Disobedient at home; Disrupts class discipline). In the current study, we used standardized scores (*T*-scores) to identify students who have scores above the borderline threshold (*T*-score = 60). A *T*-score of 59 or lower indicates that the child is not exhibiting clinically significant levels of externalizing behaviors. A *T*-score between 60 and 64 suggests that the child is exhibiting mild levels of externalizing behaviors and is at risk of developing significant externalizing behaviors. A *T*-score of 65 or higher indicates that the child is exhibiting clinically significant levels of externalizing behaviors. The CBCL and TRF showed significant discrimination between clinically referred and non-referred children (Achenbach & Rescorla, 2001) and demonstrated significant associations with other behavior rating scales, such as the Behavior Assessment System for Children (BASC; Doyle, Ostrander, Skare, Crosby, & August, 1997). Internal consistency was acceptable for both parent and teacher reports (Cronbach’s alpha, .89 and .95, respectively).

#### Social Skills

Social skills were assessed using both the teacher and parent versions of the *Social Skills Rating System* (SSRS-T; SSRS-P; Gresham & Elliot, 1990). Teachers and parents were asked to rate each item based on their perceived frequency of target behavior using a 3-point scale from 0 = “*never*” to 2 = “*very often*”. In the present study, three of the four subscales (Assertion, Self-Control, and Cooperation) were used. Because the Responsibility subscale is only included in parental SSRS, it was not included in these analyses. We used the sum of raw scores from the Cooperation, Assertion, and Self-Control subscales, each consisting of ten items. Higher sum scores indicate better social skills. The teacher and parent versions include both identical items (e.g., Uses time appropriately while waiting for help, Makes friends easily, Receives criticism well,) and items tailored to each context. The Cooperation assesses cooperative behaviors facilitating social success (e.g., Attends to your instructions, Volunteers to help family members with the task.). The Assertion subscale measures behaviors of initiating social interactions or expressions of opinion (e.g., Initiates conversations with peers, Invites others to your home.). Self-Control measures behaviors of inhibiting impulses or negative behavior (e.g., Controls temper in conflict situations with peers, Speaks in an appropriate tone of voice at home.) The SSRS was normed based on a large and national sample of 4170 children ages 3 to 18. This instrument is a well-established scale with robust validation across various populations, demonstrating strong internal consistency and validity (Gresham & Elliott, 1990). Given that the data collection intervals are confined to the middle childhood developmental stage and comprehensive validation evidence of this scale, we did not conduct tests for measurement invariance across the four time points. Parent and teacher-reported social skills at each time point showed sound internal-consistency reliabilities (Cronbach’s alphas, .76–.95).

#### Covariates

Child sex, race/ethnicity, and socio-economic status (SES) were included as time-invariant covariates in the multi-group latent growth model to account for individual differences in initial levels and growth of social skills. Parents reported their child’s sex and race/ethnicity when the child was one month old. Child sex was dummy coded (female = 0, male = 1). Child race/ethnicity included American Indian/Alaska Natives, Asian/Pacific Islander, Black, White, and Other. Given the limited variability in racial/ethnic categories, we coded race/ethnicity as a dichotomous variable (non-White = 0, White = 1). SES was measured by the income-to-needs ratio of third grade. The total family income was divided by the U.S. Census-based poverty thresholds for appropriate family size to obtain an income-to-needs ratio.

### Statistical analysis

Means, standard deviations, and correlations among the primary study variables were calculated using SPSS version 18. Multi-group latent growth model was conducted using MPlus Version 8. As is typical in longitudinal studies, not all children had complete data sets. Missing data were handled using Full Information Maximum Likelihood, the default in MPlus. Data analysis was conducted in three parts. First, two separate latent growth models were fit to the whole sample. One model used teacher-reported social skills, and the other used parent-reported social skills to examine whether children showed growth in social skills across school and home contexts. For both latent growth model analysis, we tested the no growth model, linear growth model, latent basis model, and quadratic growth model, and compared their fits to identify the model that best fits the data. Second, three time-invariant covariates (child sex, race/ethnicity, SES) were added to the latent growth model. Nonsignificant paths from covariates to latent intercept and slope, and covariance between covariates were fixed to zero not to estimate. Significant covariates were freely estimated. This process was done both for the teacher-reported and parent-reported social skills latent growth model. Third, four groups were created based on the borderline cut point (*T*-score = 60) of CBCL externalizing behavior in third grade. Although the “clinical” range (*T*-score =65+) highlights the most significant areas of need for behavioral intervention, the importance of attention toward behaviors in the “borderline” (*T*-score = 60–64) should not be overlooked. Behaviors within the borderline level point to important areas for early intervention, allowing for resources to be directed toward areas of emerging concern before they escalate to a more severe presentation. Using the CBCL borderline, four groups were formed as follows: *No Concern* (NC) group had externalizing behavior rated as typical level or lower than the borderline by both parents and teachers. *Parent Concern (PC)* group had externalizing behavior rated above the borderline by parents but typical by teachers. In contrast, *Teacher Concern* (TC) group had externalizing behavior rated as typical by parents but above the borderline by teachers. *Both Concern* (BC) group had externalizing behavior rated above the borderline by both parents and teachers. Finally, we conducted two separate multigroup latent growth model analyses, one using teacher-reported and the other using parent-reported social skills. Each multigroup latent growth model assessed whether initial levels and growth rates differed across the four behavioral groups. We first estimated a fully constrained model in which latent means, variances, covariances, and residual variances were held equal across groups, and compared it to less constrained models allowing these parameters to vary. During this stage, covariate effects (child sex, race/ethnicity, and SES) were held constant across groups. After identifying the best-fitting model, we estimated an additional model allowing covariate effects to vary across groups, and compared model fit again. Additionally, Wald tests were conducted to determine whether specific growth parameters (e.g., intercepts, slopes) significantly differed between groups.

## Results

### Descriptives

Descriptive statistics of primary variables are presented in Table 1. For both teacher and parent reports, the average *T*-scores for externalizing behavior in third grade were comparable to those for the normed sample (Achenbach, 1991a). Nevertheless, 11.3% of children were rated by parents as having externalizing behaviors above the borderline (*T*-score ≥ 60) and 18.6% of children were rated by teachers as exhibiting externalizing behaviors above the borderline (*T*-score ≥ 60). Average raw scores on social skills (cooperation, assertion, and self-control) approximated the normed mean at all four time points (Gresham & Elliot, 1990). We formed four groups based on the agreement or disagreement between teachers and parents regarding externalizing behaviors, using the CBCL borderline as a criterion. For a majority of students (NC group, 75%) parents and teachers agreed that the child’s externalizing behaviors were not notably concerning (see Table 2). However, 21% of participants fell into discrepancy groups, where one informant (either the parent or teacher) expressed concern that the child’s externalizing behaviors were above the borderline, while the other did not (PC: 7%, TC: 14%). Students in the discrepancy groups were more likely to be non-White and from low-SES backgrounds compared to those in the NC group. Additionally, 4% of students were placed into the BC group, where both parents and teachers expressed concern that the child’s externalizing behaviors significantly exceeded the borderline threshold.

### Overall social skill growth: baseline model selection

In the first step, we compared the no growth model, linear growth model, latent basis model, and quadratic growth model using a whole sample to examine whether social skills change during middle childhood shows growth and if so, whether this growth is strictly linear or nonlinear. The comparison of nested models for teacher and parent reports on social skills is presented in Table 3. The model comparison for teacher reports on social skills indicated that the linear growth model had a significantly better fit compared to the no growth model. Following that, the linear growth model was compared separately with the latent basis model and the quadratic model. The chi-square difference test indicated that the linear growth model was not significantly different from either of the two models. Therefore, we concluded that the linear growth model, which is parsimonious, represents the data appropriately. The same procedure was applied to parent-reported social skills, and the results indicated that the linear growth model was the best fit for the data. In the second step of the analysis, the three covariates (family SES, child race, and child sex) were included in each of the linear growth models. For both models, all three covariates were found to be significantly associated with the intercept of social skills but not with the slope (see Table 4). The covariance between the covariates was not significant, except for the covariance between SES and race. Non-significant paths were fixed to zero and not estimated and any significant paths were allowed to be freely estimated in the baseline model (see Fig. 1). In the linear growth models of both teacher and parent-reported social skills, significant intercept means and variances were observed, indicating that third graders generally possess levels of social skills significantly greater than zero and that these levels vary substantially across individuals. However, the slopes were not statistically significant for either report type (see Table 4), suggesting that, on average, there is no consistent linear progression in social skills development across the cohort. Nevertheless, the significant variance in the slopes for both models highlights considerable individual differences in the trajectories of social skills development. These findings suggest that while a general trend of growth in social skills is not evident for the entire group, there are notable variations in growth patterns among different subgroups or individual children.

### Model specifications for multi-group latent growth model

Two multigroup latent growth models of teacher- and parent-reported social skills were conducted using four groups (NC, PC, TC, and BC) formed based on third grade externalizing behaviors. For both models, the analysis employed the baseline model established in steps 1 and 2 (see Fig. 1). Nested models were tested incrementally, gradually freeing latent means, variances, covariances, and residual variances across groups (see Table 5). Covariates were initially constrained to remain equal across all four groups in each model. Model 1 represented a fully constrained group-invariant model, where latent means, variances, covariances, and residual variances were held constant across groups. In Model 2, the latent means for intercept and slope were freed. Model 3 allowed additional flexibility by freeing the variances and covariances of the intercept and slope. Model 4 further relaxed constraints by freeing the residual variances. In the analysis of teacher-reported social skills, Model 4 exhibited significantly enhanced fit compared to other models; however, both Models 3 and 4 were discounted due to the presence of negative variance errors, which indicate an overestimation of model parameters (Kolenikov & Bollen, 2012). Thus, Model 2, which achieved a satisfactory fit (RMSEA = .06, CFI = .92), was ultimately selected. For purposes of model comparison, Model 2 initially constrained covariates equally across groups; these constraints were later relaxed in Model 5 to assess the differential impact of covariates across groups. Comparative analyses between Models 2 and 5 indicated no significant differences, thus confirming Model 2 as the final model in teacher-reported social skills. In the analysis of parent-reported social skills, the same analytical approach was applied, and Model 2 demonstrated the best fit. Similar to the teacher-reported model, covariates were freely estimated in Model 5 and compared to Model 2. As no significant differences were observed between Models 2 and 5, Model 2, being the more parsimonious model, was retained for analyzing linear growth in parent-reported social skills.

### Teacher-reported social skills trajectory across groups

Using Fig. 1 as the baseline model, we examined whether the growth intercepts and slopes of social skills from third to sixth grade differed across the four groups. The analysis revealed significant mean intercepts for all groups, indicating distinct initial social skill levels at third grade (Time 1). Specifically, the NC group exhibited the highest baseline social skills, followed by the PC, TC, and BC groups (see Table 6 and Fig. 2). Pairwise comparisons using the Wald test revealed significant differences in initial social skill levels across most group pairs. Notably, all comparisons between the NC group and the other groups were significant, underscoring the greater social skills in students for whom parents and teachers concurred on a typical level of externalizing behavior. However, the comparison between two groups (TC and BC) where teachers reported externalizing behaviors above the borderline cutoff at third grade did not show significant differences in social skills at third grade (Wald (1) = 0.71, *p* = .400). For the slope, the TC and BC group demonstrated a significant linear increase in social skills from third to sixth grade whereas the PC and NC groups showed no significant change. Specifically, the TC group exhibited a significant increase in social skills and the BC group showed a trend toward significance. However, the magnitude of slopes for these two groups were not significantly different (Wald (1) = 2.85, *p* = .091). The covariances and variances were constrained to be equal across all four groups. The covariance between the intercept and slope was not significant, suggesting no evidence that initial social skill levels at third grade were related to the growth rate of social skills. However, the variances of both the intercepts and slopes were significant, indicating variability in initial levels and growth trajectories of social skills within each group.

### Parent-reported social skills trajectory across groups

Adopting a multi-informant approach, we conducted the same analysis for parent-reported social skills to evaluate whether the growth intercept and slope varied across the four groups in home contexts (see Fig. 3). The intercepts for all groups were significant, reflecting distinct initial levels of social skills at third grade. The NC group, where both parents and teachers agreed on typical levels of externalizing behavior, demonstrated the highest social skills at third grade (Time 1), followed by the TC, PC, and BC groups (see Table 6). Pairwise comparisons using the Wald test indicated that the NC group had significantly greater social skills at third grade compared to all other groups. Most pairwise differences were statistically significant, except for the comparison between the PC and BC groups—two groups in which parents reported externalizing behaviors above the borderline cutoff at third grade (Wald (1) = 0.87, *p* = .351). The slopes for all groups were non-significant, indicating no detectable linear change in parent-reported social skills from third to sixth grade across the groups. Covariances and variances were constrained to be uniform across all groups. The covariance between the intercept and slope was not significant, suggesting no association between initial social skill levels and the rate of change. However, the variances of both the intercepts and slopes were significant, reflecting variability in the baseline levels and growth trajectories of social skills within each group.

## Discussion

This study explored how parent-teacher agreement and disagreement regarding externalizing behaviors predict subsequent social skill trajectories as reported by teachers and parents. Building on existing research that examines the associations between early behavioral problems and social development (e.g., Berry & O’Connor, 2010; Racz et al., 2017), this study further investigated the role of agreement and disagreement on behavioral problems between parents and teachers who serve as the two primary informants in behavioral assessments during middle childhood. Discrepancies between parents and teachers should prompt nuanced observations of developmental contexts; however, differences are often overlooked, with assessments relying on a single informant’s perspective or treating discrepancies as measurement errors (von der Embse & De Los Reyes, 2024). This oversight might compromise children’s developmental potential by preventing precise identification and appropriate support of their needs. To investigate the potential influence of informant discrepancies on social skill development, this study analyzed parent-teacher agreements and discrepancies regarding externalizing behaviors in third grade, and examined how these discrepancies predicted teacher- and parent-reported social skills trajectories from third to sixth grade.

## Overall social skill trajectories: individual variations in developmental patterns

The analysis of overall trajectory patterns across the sample revealed no significant growth in social skills from third to sixth grade, but significant variance in growth rates for both teacher- and parent-report models, indicating substantial heterogeneity across participants. This pattern aligns with previous research demonstrating children follow different developmental paths, beginning their growth at various starting points and developing social skills at distinct rates during middle childhood (Cote et al., 2002; DiDonato, 2014). The non-significant slope for the entire participant group across two models likely reflects the offsetting effects of contrasting individual trajectories, where some children exhibited growth, others showed decline, and some maintained stable levels of social skills over time. For example, Sorlie et al. (2021) identified three distinct trajectory groups among children followed from fourth to seventh grade: one characterized by stable average social skills, another with high initial levels followed by a decline, and a third with low initial levels followed by an increase. Additionally, several studies have demonstrated curvilinear growth in social skills and prosocial behaviors during middle childhood (Berry & O’Conner, 2010; Troncoso & Humphrey, 2021). Although our study found that a linear growth model provided a better fit than non-linear alternatives for both teacher and parent ratings, the observed non-significant slope may be attributable to these mixed developmental patterns in social skills development. This finding underscores the need for further investigation into factors contributing to individualized developmental trajectories. Multiple studies have identified individual and contextual factors - such as early behavioral problems, gender, race/ethnicity and parenting characteristics - that shape these diverse developmental pathways during middle childhood (Berry & O’Connor, 2010; Hajovsky et al., 2022; Hajovsky, Chesnut, Sekula, & Olsen, 2024). Berry and O’Connor (2010) found that early internalizing behaviors at 54 months predict less adaptive social skill trajectories through sixth grade, while Hajovsky et al. (2024) reported that racial/ethnic differences influence trajectories, with Black students exhibiting distinct patterns of decline in social skills. Furthermore, Frogner et al. (2022) demonstrated how child gender and temperament characteristics interact to predict developmental trajectories, thus highlighting the complexity of social skill development. Additional research into social skills and the driving forces behind distinct developmental trajectories across settings would enhance our understanding of these developmental processes.

## Informant discrepancies between parents and teachers on externalizing behavior

Our findings indicate that for 75% of students, both parents and teachers agreed that externalizing behaviors fell within the normal range (No Concern), while for 4%, both informants agreed that behaviors exceeded the borderline threshold, indicating emerging concern about externalizing behavior (Both Concern). However, for 21% of students, parent and teacher evaluations diverged, with one informant reporting behaviors above the borderline threshold and the other within the normal range (Parent Concern 7%, Teacher Concern 14%). Although parents generally reported lower externalizing behavior scores than teachers, some students were identified with elevated behaviors only by parents (7%), highlighting the unique perspective each informant provides. This finding aligns with previous studies on informant discrepancies, which suggest that children’s behavioral problems often manifest differently across settings (De Los Reyes et al., 2015; Stone, Speltz, Collett, & Werler, 2013). A distinct aspect of our study is the use of the borderline threshold as a criterion for examining informant discrepancies. The borderline range, an intermediate zone between normal and clinical ranges, serves as an early warning system that necessitates careful monitoring and attention (Achenbach & Rescorla, 2001). Our results suggested that for 21% of students, parents and teachers held divergent perspectives on whether the students’ externalizing behaviors crossed the borderline threshold, thereby warranting clinical concerns. This finding supports the importance of a multi-informant approach for early detection of behavioral concerns and enabling timely interventions to support and improve children’s social behaviors. However, De Los Reyes et al. (2022) pointed out that decisions about referrals and interventions are often based on the primary informant – teachers for referral to school-based services and parents for referral to community or private – which can result in behavioral challenges being overlooked or minimized when those challenges manifest primarily in one context but not the other.

One notable finding is that students in the discrepancy groups (PC, TC) were disproportionately non-White and from lower socioeconomic backgrounds compared to those in the NC group. This concurs with previous research showing that factors such as race/ethnicity and parental socioeconomic status often exacerbate discrepancies between parent and teacher evaluations of children’s behaviors and mental health concerns, though the underlying mechanisms remain unclear (Kuhfeld et al., 2023; Stone et al., 2013). Cultural differences in expectations, social norms, and values associated with race/ethnicity and socioeconomic status might contribute to this increased parent-teacher disagreement. While parents may hold diverse views on what constitutes appropriate and socially competent behaviors, school-wide expectations are frequently set without the input of diverse families, thereby reflecting the values of the dominant culture (Bal, 2018). Given that the NICHD SECCYD sample is predominantly composed of White, middle-class families, it is likely that informant discrepancies are even more prevalent in more socioeconomically and culturally diverse educational settings. These findings underscore the importance of systematically incorporating multi-informant discrepancies into behavioral assessments, particularly in increasingly diverse educational settings.

## Informant discrepancies in externalizing behavior and social skills development

In both parent- and teacher-reported models, children in the NC group were reported to display the highest social skills, and those in the BC group the lowest, from third to sixth grade. Children in the discrepancy groups (PC, TC) fell between these two extremes, with these patterns remaining stable through sixth grade. Agreement between parents and teachers on externalizing behavior appears to be a strong predictor of social skill development, with the two agreement groups reporting the highest (NC) and lowest (BC) stable social skills trajectories. This finding supports converging operations, wherein consistent reports across multiple informants serve as particularly reliable behavioral indicators (De Los Reyes et al., 2013). That is, a consensus between parents and teachers regarding a child’s externalizing behaviors may be a robust indicator of subsequent social development.

For parent-teacher discrepancies, we found that children in the discrepancy groups, although reported to have lower social skills than those in the NC group, were consistently reported as having higher social skills compared to peers in the BC group. This pattern suggests that informant discrepancies themselves may not function as an additional risk factor impeding development—a finding that differs from prior studies demonstrating the unique role of informant discrepancies in explaining the development of psychopathology (Laird & De Los Reyes, 2013) or in hindering intervention effectiveness (Goolsby et al., 2018; Hennig et al., 2017). The observed differences could be attributed to our methodological approach, which utilizes informant discrepancies as grouping variables rather than analyzing them directly.

However, our analysis indicates that informant discrepancies can provide insights into contextual risks that shape children’s social skill development. Specifically, children in the discrepancy groups were reported to have lower social skills compared to the NC group across home and school contexts from third through sixth grade, even in contexts where minimal concerns about externalizing behaviors were reported. According to the bioecological model (Bronfenbrenner & Ceci, 1994), which posits that human development is shaped by dynamic interactions across multiple contexts, our results suggest that externalizing problems identified in one setting may extend their negative influence, affecting children’s behavior in other contexts and their overall social skill development. This finding corroborates the developmental cascades by illustrating how externalizing behaviors in one context may impact social skills across multiple contexts. The potential of such cross-context cascades underscore the need for a holistic approach to behavioral challenges and the systematic integration of diverse perspectives in both assessment and intervention strategies.

## Teachers as proactive agents and parental challenges in social skill development

Our study identified varying growth patterns in social skills between the teacher-reported and parent-reported models. In the teacher-reported model, students in both groups where teachers reported externalizing concerns (TC and BC) demonstrated significant gains in reported social skills from third to sixth grade. Conversely, in the parent-reported model, no group showed significant social skill growth. These findings suggest that significant improvements in social skills at school were observed among students whose teachers identified them as displaying high externalizing behavior. The observed differential growth patterns might reflect the role and capacity of teachers in supporting students’ social-behavioral development. Teachers’ professional training and daily exposure to diverse students enable them to recognize developmental variations and identify students requiring additional behavioral monitoring and support. They are also uniquely positioned to implement behavior management strategies or social-emotional learning programs within the structured school environment, which can foster improvements in students’ social skills (Domitrovich et al., 2017). Additionally, teacher’s perspectives are regularly used within the school settings to refer students to more intensive interventions, such as counseling or behavioral interventions, often carrying more weight than parent reports in decisions about accessing services (Stiffman, Pescosolido, & Cabassa, 2004). Such targeted and intensive interventions in school settings are often more systematic and immediate compared to what is typically available in the home context.

Parents, however, often encounter distinct challenges in addressing their children’s behavioral difficulties. They may struggle to recognize the severity of behaviors due to limited knowledge of normative development or may acknowledge the need for support but face significant barriers in accessing resources. For example, Meng and Wiznitzer (2024) highlighted that parents frequently do not receive mental health services for their children due to problems getting an appointment, high costs, or limited availability of services in their area. Additionally, cultural differences in interpreting behavior and the stigma associated with seeking external support can further complicate their ability to obtain appropriate assistance for their children (Hansen, Telléus, Mohr-Jensen, & Lauritsen, 2021). Von der Embse and De Los Reyes (2024) suggests that systematic integration of families into school-based mental health services may help address these barriers since family involvement in the decision-making process not only provides context-sensitive data for assessments and interventions but also creates pathways for more equitable resource access. This integrated approach could effectively leverage teachers’ proactive role in behavioral support while facilitating meaningful family engagement in the intervention process.

## Practical implications

The present findings suggest several implications for educational and clinical practice. First, our results demonstrate that informant discrepancies in externalizing behaviors might serve as a meaningful indicator of contextual risk in children’s social development rather than mere measurement error. While the value of multi-informant approaches in behavioral assessment has been well-established (Hunsley & Mash, 2018), practical constraints often lead to reliance on single-informant data in applied settings (De Los Reyes et al., 2022). Our findings underscore that relying on a single informant may overlook developmental risks, as children identified with elevated externalizing behaviors in even one context were reported to demonstrate consistently lower social skills across settings. These results highlight the potential need for practitioners to incorporate both parent and teacher perspectives into assessment practices, and for teachers to enhance communication with parents regarding children’s behavioral concerns. Second, our findings call for a reconceptualization of how informant discrepancies in evaluations of child behavior are interpreted in applied settings. Rather than viewing disagreements between parents and teachers as obstacles to assessment, these discrepancies should be understood as windows into the context-specific factors shaping children’s behavior. When divergent observations emerge between home and school contexts, practitioners can use these differences to identify specific environmental conditions that may support or challenge a child’s social functioning. Despite significant advances in understanding informant discrepancies in clinical psychology over the past two decades, a substantial research-practice gap remains (De Los Reyes et al., 2022; Martel et al., 2017), suggesting the pressing need to translate these empirical insights into practical applications. Third, our findings suggest the importance of strengthening home-school connections and collaborative communication between parents and teachers. Our results revealed that while some children’s social skills were reported to improve over time by teachers, these gains were not consistently reflected by parents. This pattern suggests that positive developments occurring in school settings may not be effectively generalized to home environments, or that such changes might not be readily observable to parents. These findings point to the value of enhanced parent-teacher communication to ensure both informants are aware of children’s behavioral patterns across contexts.

## Limitations and future directions

There are several limitations to consider when interpreting the results of this study. First, the current sample, drawn from the NICHD SECCYD, represents a relatively low-risk population. Consequently, most participants demonstrated normative levels of externalizing behaviors and social skills, which may have limited variability compared to other samples. The creation of four groups based on a clinical borderline of externalizing behavior resulted in relatively small sample sizes for the PC, TC, and BC groups, particularly due to the limited representation of high-risk participants. Future studies should aim to include more diverse samples to better understand the predictive value of informant discrepancies in social skill development. Second, this study operationalized discrepancies based on whether participants’ externalizing behaviors fell above or below the borderline clinically significant cutpoint. This approach is practical for identification and service provision, as children scoring above the borderline are more likely to be identified and referred for services, whereas those scoring below the threshold are less likely to receive intervention. However, this binary approach does not capture scenarios such as large discrepancies between parent and teacher ratings within the normal range of scores that are marginally below the borderline. It can also overestimate discrepancies when scores differ by just one point around the borderline threshold—an outcome that might be easily influenced by how individual raters interpret and use the scale. Exploring alternative methods for defining and analyzing discrepancies, such as person-centered approaches and polynomial regression techniques (Laird & De Los Reyes, 2013), would benefit future research by providing a more comprehensive understanding of how discrepancies influence social behavior development. Third, longitudinal measurement invariance testing was not conducted for the parent and teacher reports of social skills. We used the Elementary Form of the SSRS, a well-established and widely used instrument standardized for students from Kindergarten through Grade 6. Given prior evidence of longitudinal measurement invariance for this instrument across elementary grades (Caemmerer & Keith, 2015; Hajovsky et al., 2024), we relied on existing validation evidence. Nonetheless, formal invariance testing could further strengthen our interpretation of developmental changes in social skills. Another limitation is that teacher reports were likely provided by different teachers each year, whereas parent reports were likely completed by the same individual over time. Difference in informant continuity may have influenced the observed trajectories, as parents may rely on more trait-based evaluations that are less sensitive to behavioral changes, while teachers may be more attuned to context-specific behaviors within each school year. Consequently, observed different trajectories for teacher- and parent-reported social skills may partly reflect these structural differences in rating approaches rather than solely representing true developmental change.

## Conclusion

The findings from this study suggest an intricate relationship between parent-teacher discrepancies in externalizing behaviors and subsequent social skills development: while informant discrepancies were not systematically associated with social skill development as an independent risk factor, they may serve as indicators of contextual risks that influence students’ social skill trajectories. These results support the importance of integrating informant discrepancies in the assessment of child behavior, both in developmental research and applied practice, to enhance our ability to understand and support children’s social skill development. Despite the central roles of parents and teachers as primary informants, the developmental implications of their discordant behavioral evaluations have received limited attention in child development research. This study suggests the need for further research to investigate how agreements and discrepancies between parent and teacher evaluations can be leveraged to enhance assessments and interventions, offering deeper insights into children’s developmental processes.

## Figures and Tables

**Fig. 1.**
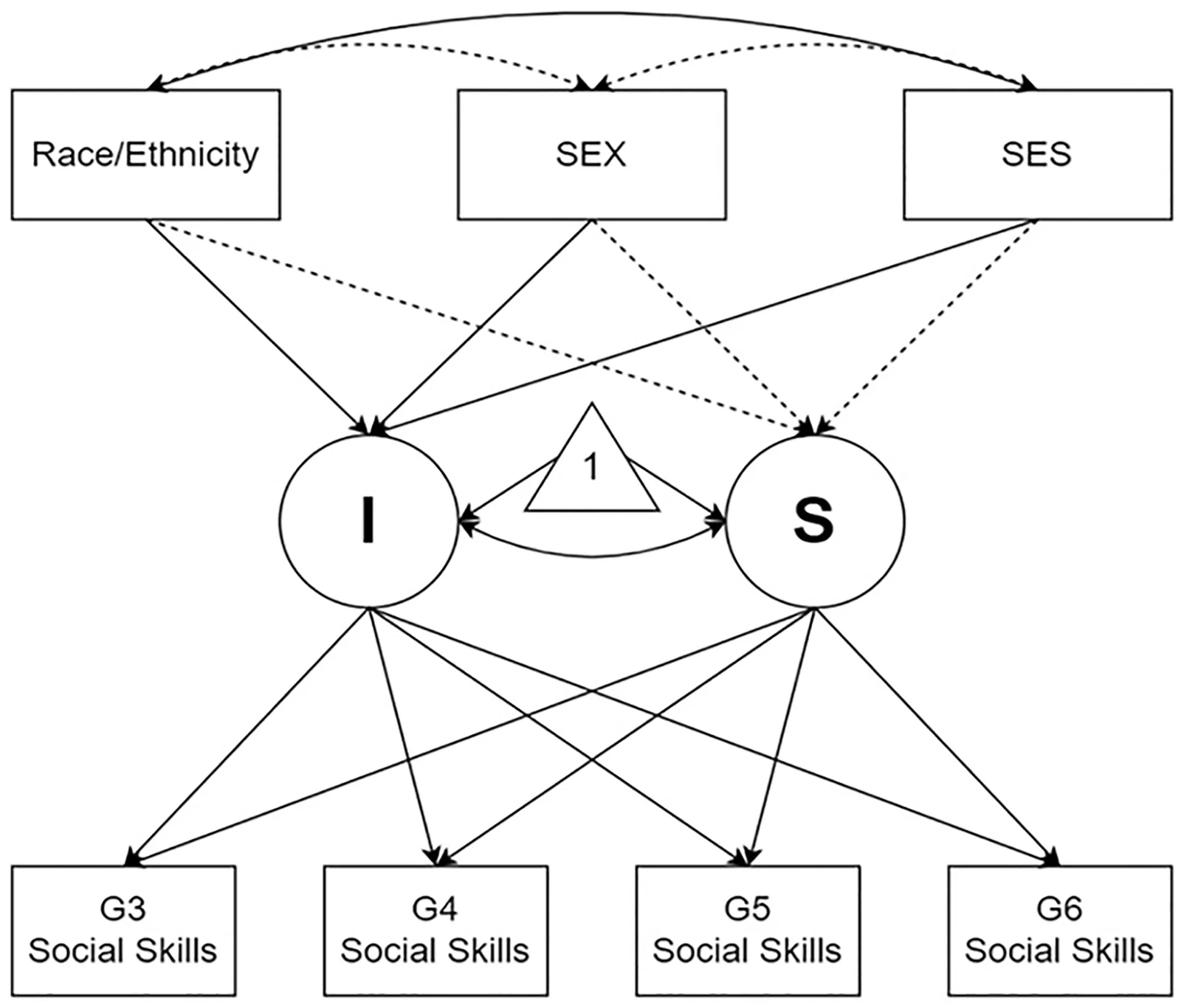
Baseline Linear Growth Model of Social Skills From Grades 3 to 6 With Time Invariant Covariates. *Note*. Solid lines represent significant paths. Dashed lines indicate non-significant paths. G3–G6 = Grades 3–6.

**Fig. 2.**
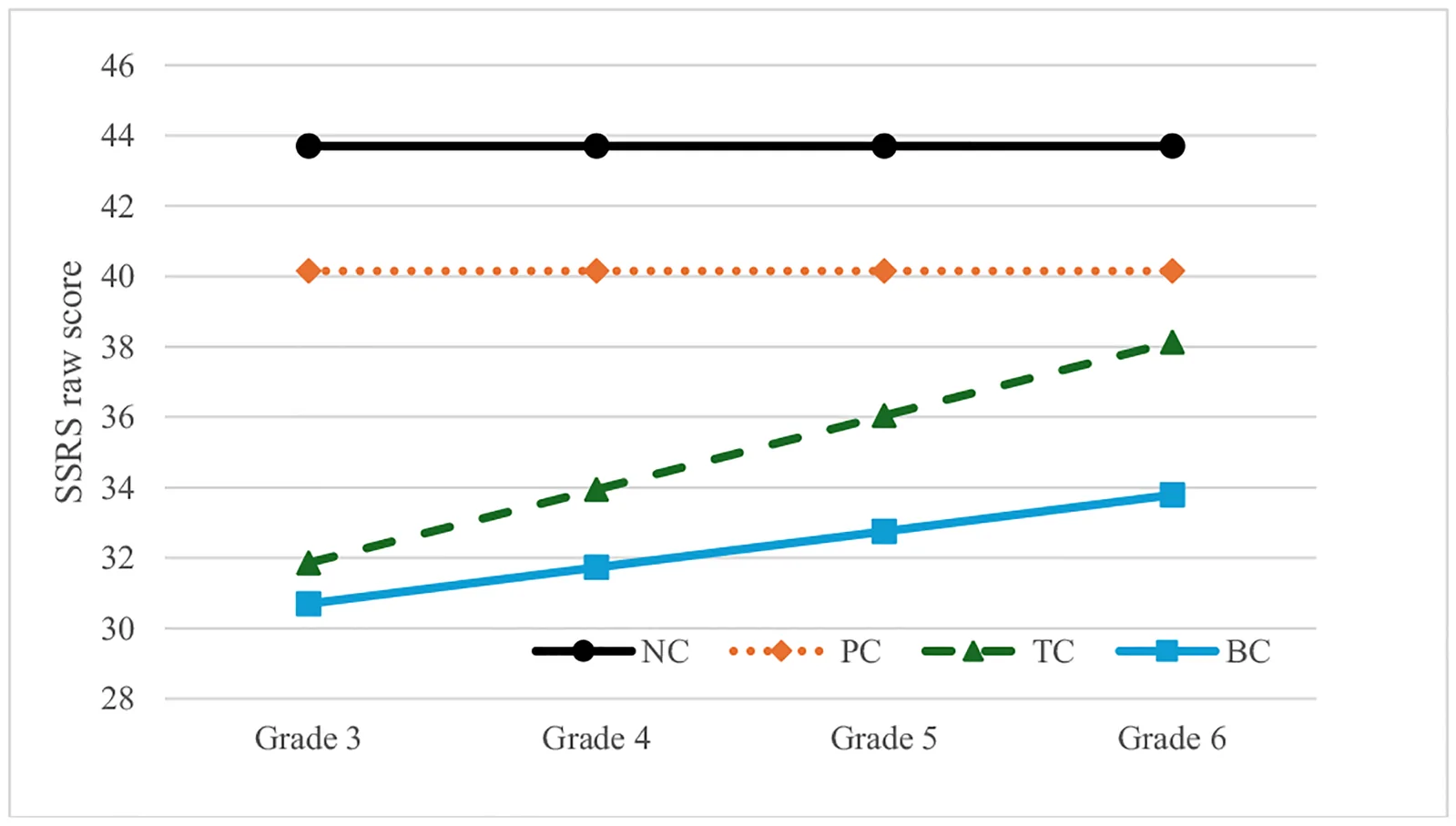
Teacher-Reported Social Skills Trajectories From Third to Sixth Grade Across Four Groups. *Note*. NC = No Concern. PC = Parent Concern. TC = Teacher Concern. BC = Both Concern. SSRS = *Social Skills Rating System*. This figure displays the trajectory of social skills based on the estimated means from the final linear growth model, not the sample mean. The SSRS raw score is the sum of subscale scores for Cooperation, Self-control, and Assertion, which can range from 0 to 60.

**Fig. 3.**
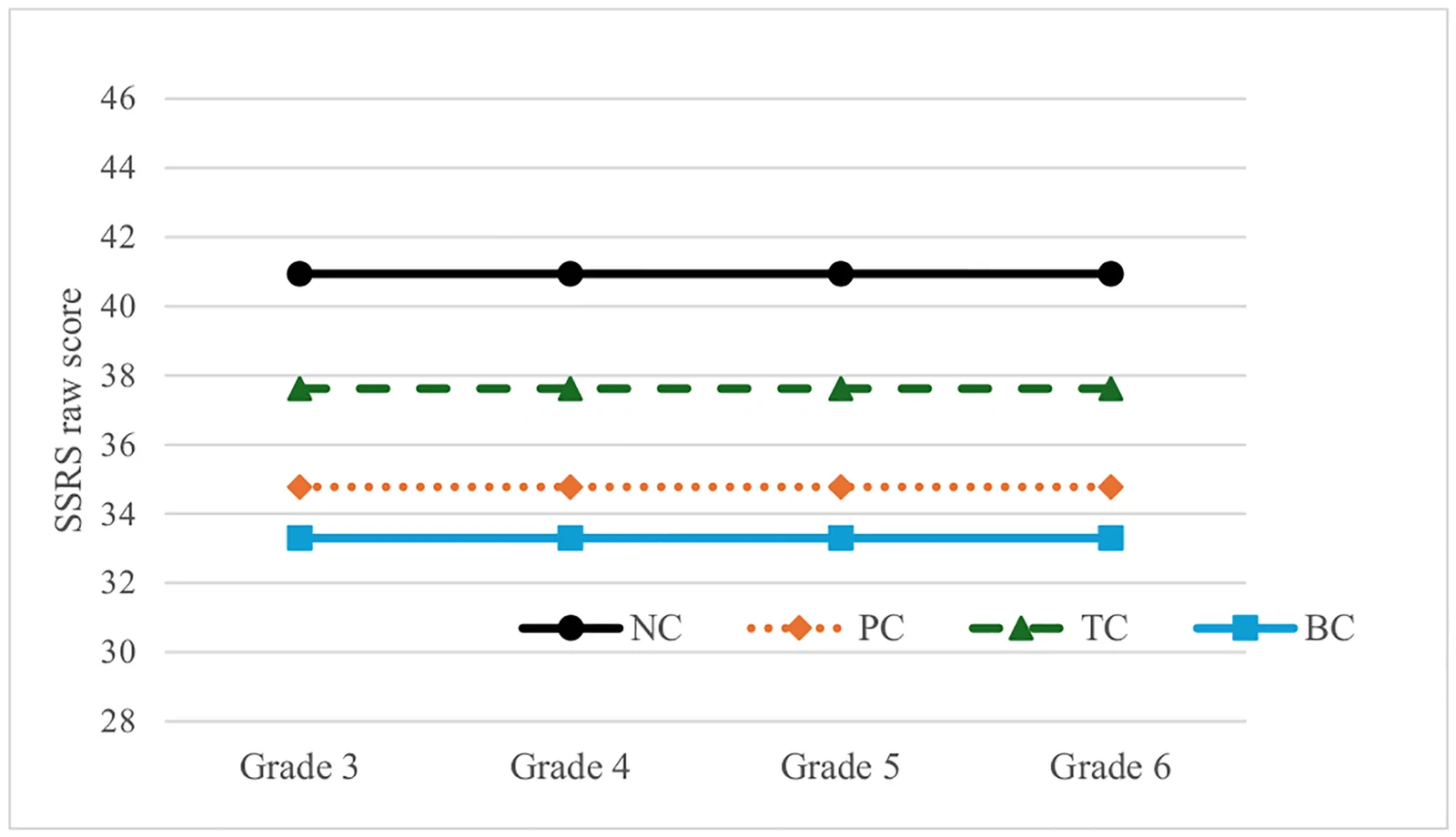
Parent-Reported Social Skills Trajectories From Third to Sixth Grade Across Four Groups. *Note*. NC = No Concern. PC = Parent Concern. TC = Teacher Concern. BC = Both Concern. SSRS = *Social Skills Rating System*. This figure displays the trajectory of social skills based on the estimated means from the final linear growth model, not the sample mean. The SSRS raw score is the sum of subscale scores for Cooperation, Self-control, and Assertion, which can range from 0 to 60.

**Table 1:** Descriptive Statistics and Correlations for Primary Study Variables.

	1	2	3	4	5	6	7	8	9	10	11	12	13
Teacher reports													
1. G3 EB	1.00												
2. G3 SS	−.61***	1.00											
3. G4 SS	−.46***	.56***	1.00										
4. G5 SS	−.38***	.48***	.55***	1.00									
5. G6 SS	−.34***	.47***	.50***	.57	1.00								
Parent reports													
6. G3 EB	.41***	−.30***	−.27***	−.27***	−.21***	1.00							
7. G3 SS	−.30***	.37***	.34***	.31***	.25***	−.39***	1.00						
8. G4 SS	−.26***	.30***	.34***	.30***	.35***	−.33***	.76***	1.00					
9. G5 SS	−.23***	.29***	.31***	.33***	.30***	−.34***	.71***	.76***	1.00				
10. G6 SS	−.26***	.31***	.32***	.31***	.33***	−.35***	.66***	.73***	.75***	1.00			
11. Sex (male)	.01	−.21***	−.23***	−.20***	−.29***	−.04	−.15***	−.16***	−.10*	−.14***	1.00		
12. Race (White)	−.21***	.19***	.24***	.19***	.22***	−.07*	.07	.17***	.08*	.08	.01	1.00	
13. SES	−.17***	.17***	.24***	.23***	.23***	−.21***	.22***	.23***	.23***	.25***	.00	.22***	1.00
*M (%)*	51.4	43.3	43.5	43.8	43.9	47.5	42.0	42.1	41.7	42.0	49%	82%	4.4
*SD*	9.2	10.3	9.9	10.1	10.2	9.9	7.4	8.3	7.5	8.2	0.5	0.4	3.7
*Min*	39	8	11	11	9	30	19	14	16	10	0	0	0.1
*Max*	87	60	60	60	60	78	60	60	60	60	1	1	26.6
*N*	927	919	816	814	739	927	671	615	658	632	927	927	886

*Note*. G3–G6 = Grades 3–6. EB = Externalizing Behavior. SS = Social Skills

* *p* < .05.

*** *p* < .001.

**Table 2:** Group Membership Based on Parent and Teacher Reports on Externalizing Behavior (CBCL).

	No Concern	Parent Concern	Teacher Concern	Both Concern
*N* (%)	694 (75%)	61 (7%)	128 (14%)	44 (4%)
Description	Agreement Low Externalizing Behavior	Disagreement Parent > Teacher	Disagreement Parent < Teacher	Agreement High Externalizing Behavior
Parent-reported Externalizing Behavior	Normal	Above Border	Normal	Above Border
*M*	44.40	64.89	49.23	66.34
*SD*	7.65	4.25	7.35	3.93
Teacher-reported Externalizing Behavior	Normal	Normal	Above Border	Above Border
*M*	47.88	51.43	65.05	66.89
*SD*	6.26	5.80	5.84	9.20
Race/ethnicity (% White)	86%	82%	70%	66%
Sex (% Girl)	51%	59%	48%	59%
Income to Needs Ratio	4.7	3.7	3.7	2.8

*Note*. CBCL = *Child Behavior Checklist*. Normal refers to externalizing *T*-scores ranging from 0 to 60. Above Border indicates *T*-scores exceeding the borderline threshold of 60.

**Table 3:** Model Comparison for Establishing Baseline.

Model	*χ* ^2^	*df*	Model comparison	*Δχ* ^2^	*Δdf*	RMSEA	CFI
Teacher-reported social skills							
No growth model	34.01	11				.05	.98
Linear growth model	7.42	8	No growth vs Linear	26.59***	3	0	1
Latent Basis model	2.88	6	Linear vs Latent basis	4.54_ns_	2	0	1
Quadratic growth model	4.58	4	Linear vs Quadratic	2.84 _ns_	4	.10	1
Parent-reported social skills							
No growth model	55.56	11				.07	.97
Linear growth model	26.70	8	No growth vs Linear	28.9***	3	.05	.99
Latent Basis model	26.15	6	Linear vs Latent basis	0.55_ns_	2	.07	.99
Quadratic growth model	20.57	4	Linear vs Quadratic	5.58 _ns_	4	.07	.99

*Note*. CFI = Comparative Fit Index. RMSEA = Root Mean Square Error of Approximation. _ns_ = not significant.

*** *p* < .001.

**Table 4:** Time Invariant Covariates and Linear Growth Model of Social Skills (SSRS).

	Teacher-reported Social Skills		Parent-reported Social Skills	
	Estimate	*SE*	Estimate	*SE*
Covariates				
Child sex (male) → Intercept	−4.18***	0.60	−2.13***	0.54
Child race (White) → Intercept	4.44***	0.81	1.70*	0.81
Family SES → Intercept	0.44***	0.08	0.46***	0.07
Child sex (male) → Slope	−0.29	0.24	0.05	0.17
Child race (White) → Slope	0.13	0.33	−0.10	0.26
Family SES → Slope	0.02	0.04	0.01	0.02
Child sex (male) – race (White) covariance	0.00	0.01	0.00	0.01
Child sex (male) – family SES covariance	−0.01	0.06	−0.01	0.06
Family SES – child race (White) covariance	0.32***	0.05	0.32***	0.05
Intercept				
Mean	39.62***	0.80	39.03***	0.81
Variance	50.87***	4.06	43.00***	2.95
Slope				
Mean	0.11	0.32	−0.01	0.26
Variance	3.19***	0.70	1.39***	0.30
Intercept –Slope covariance	−5.10***	1.37	−1.33	0.71

*Note*. SSRS = *Social Skills Rating System*. Unstandardized estimates are reported to facilitate interpretation. The teacher and parent versions of the SSRS use identical response scales (0 = *Never* to 2 = *Very Often*) and parallel subscale structures, allowing for meaningful comparisons across models.

* *p* < .05.

*** *p* < .001.

**Table 5:** Model Comparison for Multigroup Latent Growth Model.

Model	*χ* ^2^	*df*	Comparison	*Δχ* ^2^	*Δdf*	RMSEA	CFI
Teacher-reported social skills							
M1 (group-invariant)	476.69	112				.12	.61
M2 (latent mean freed)	180.06	106	M1 vs M2	296.63***	6	.06	.92
M3 (latent mean, covariance, variance freed)^a^	161.28	97	M2 vs M3	18.78 *	9	.05	.93
M4 (latent mean, covariance, variance, residual variance freed)^a^	142.54	94	M3 vs M4	18.84***	3	.05	.95
M5 (latent mean, covariates freed)^b^	145.98	77	M2 vs M5	34.08 _ns_	29	.06	.93
Parent-reported social skills							
M1 (group-invariant)	243.33	112				.07	.91
M2 (latent mean freed)	159.77	106	M1 vs M2	83.56***	6	.05	.97
M3 (latent mean, covariance, variance freed)	149.36	97	M2 vs M3	10.41 _ns_	9	.05	.97
M4 (latent mean, covariance, variance, residual variance freed)	141.28	94	M2 vs M4	18.49 _ns_	12	.05	.97
M5 (latent mean, covariates freed)^b^	138.84	77	M2 vs M5	20.93 _ns_	29	.06	.96

*Note*. M1-M5 = Model 1–5.

a Models 3 and 4 for teacher-reported social skills displayed negative variance errors.

b Model 5, an extension of Model 2, features covariates that have been additionally freed to compare model fit with free covariates.

_ns_ = not significant.

* *p* < .05.

*** *p* < .001.

**Table 6:** Unstandardized Estimates for Final Linear Growth Models in Social Skills (Model 2).

	Intercept			Slope			Covariance^a^ (*SE*)
	*M* (*SE*)	Wald test	Variance (SE)	*M* (*SE*)	Wald test	Variance (*SE*)	
Teacher-reported Social Skills							
NC	43.70 (0.64)***	NC > PC > TC = BC	29.50 (3.10)***	−0.18 (0.14)	TC = BC	2.57 (0.67)***	−1.51 (1.18)
PC	40.15 (1.14)***			−0.41 (0.46)			
TC	31.86 (0.85)***			2.09 (0.32)***			
BC	30.70 (1.27)***			1.03 (0.55)^t^			
Parent-reported Social Skills							
NC	40.94 (0.73)***	NC > TC > PC = BC	37.56 (2.69)***	−0.06 (0.09)	–	1.38 (0.30)***	−1.15 (0.68)
PC	34.78 (1.19)***			0.12 (0.33)			
TC	37.62 (0.96)***			0.18 (0.25)			
BC	33.31 (1.34)***			0.00 (0.41)			

*Note*. NC = No Concern. PC = Parent Concern. TC = Teacher Concern. BC = Both Concern. The Wald test assesses whether intercepts and slopes differ significantly at the level of *p* < .05.

a The covariance between child race and family SES.

t *p* < *.10*.

*** *p* < .001.

## Data Availability

The authors do not have permission to share data.
